# Co-Feeding Transmission of Tick-Borne Viruses

**DOI:** 10.3390/v18050513

**Published:** 2026-04-29

**Authors:** Sarah C. Macon-Foley, Meghan E. Hermance

**Affiliations:** Department of Microbiology and Immunology, Frederick P. Whiddon College of Medicine, University of South Alabama, Mobile, AL 36688, USA; sm2338@jagmail.southalabama.edu

**Keywords:** co-feeding transmission, tick-borne viruses, saliva-assisted transmission

## Abstract

Tick-borne viruses (TBVs) are a diverse group of arthropod-borne pathogens maintained in complex transmission cycles involving both tick vectors and vertebrate hosts. Among the known TBV transmission routes, co-feeding transmission, in which virus is transferred from an infected tick to an uninfected tick feeding on the same vertebrate host even in the absence of a detectable viremia, represents an important route that contributes to viral maintenance in nature. Although co-feeding transmission has been demonstrated across multiple vector, host, and virus combinations, the mechanisms governing this transmission route remain poorly defined. This review synthesizes current understanding of co-feeding transmission and highlights the importance of ecological and immunological factors that shape this process in nature. Specifically, we emphasize the role of the cutaneous microenvironment at the tick co-feeding site, where localized viral replication and tick salivary factors create conditions favorable for virus transfer between co-feeding ticks. We also highlight the requirements for co-feeding transmission to occur in nature and across seasons. Together, these insights support a model in which localized skin infection is a central feature of co-feeding transmission while underscoring key gaps in our understanding of the cellular and molecular mechanisms that govern this process.

## 1. Introduction

Ticks have long been recognized as vectors of diverse pathogens, including viruses. The viruses that circulate between ticks and vertebrate hosts are known as tick-borne viruses (TBVs). A number of TBVs are causative agents of human and animal diseases, posing threats to global public and veterinary health. However, vertebrate reservoir hosts have only been identified for a subset of TBVs, making it challenging to fully understand the ecology of these viruses and thus to implement appropriate control and preventative measures. Understanding the transmission cycles that enable these viruses to persist in nature is therefore important for identifying potential targets and informing strategies aimed at reducing virus transmission by ticks.

Transmission of TBVs is evaluated through two routes: vertical transmission and horizontal transmission. Vertical transmission includes: (i) transovarial transmission, where a virus is passed from an infected adult female tick to her eggs, and (ii) transstadial transmission, where a virus is maintained by an individual tick as it molts from one life cycle stage to the next [1]. Horizontal transmission occurs when a virus is passed between ticks via the vertebrate host on which they feed. Traditionally, it was accepted that ticks acquire virus during a blood meal if the vertebrate host develops an obvious and relatively high viremia (i.e., virus circulating in the host bloodstream) [2]. However, research in the late 1980s provided a nuanced picture of horizontal transmission with the discovery of nonviremic co-feeding transmission. This process enables transmission of a virus between infected and uninfected ticks feeding simultaneously on the same vertebrate host, even in the absence of detectable viremia [3,4]. Discovery of nonviremic co-feeding transmission challenged the dogma that vertebrate host viremia is required for feeding ticks to become infected [2]. Tick salivary molecules are also known to influence pathogen transmission through a process termed saliva-assisted transmission (SAT), in which salivary components modulate host immune responses at the tick feeding site and create a microenvironment favorable for pathogen establishment and localized virus replication [5,6,7]. This review highlights co-feeding transmission of TBVs, with an emphasis on the early literature that first described nonviremic co-feeding transmission, the required conditions for its occurrence, and the localized skin infection at the site of tick co-feeding that supports this unique form of transmission.

## 2. Co-Feeding Transmission of TBVs

**Figure 1:**
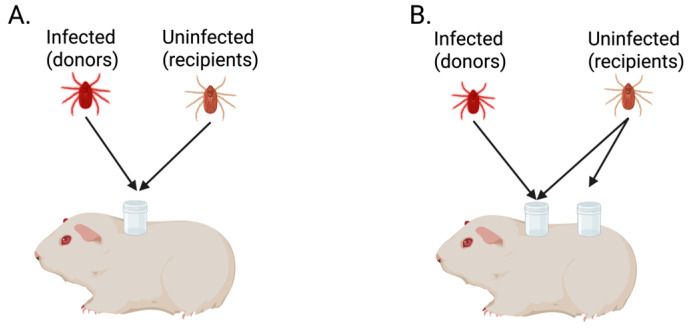
Overview of tick infestation strategy in the seminal work by [3], demonstrating nonviremic tick co-feeding transmission of virus. (**A**) Donor and recipient ticks were placed within the same feeding capsule. (**B**) Donor and recipient ticks were placed in the same feeding capsule with a second group of recipient ticks placed in a separate capsule on the same host. Created with Biorender.com (https://BioRender.com/h488wlu) (accessed on 22 March 2026).

In 1987, Jones et al. [3] were the first to challenge the prevailing belief that a detectable vertebrate host viremia is required for ticks to acquire virus while feeding. Their work investigated tick-to-tick transmission of Thogoto virus (THOV) by mimicking the natural scenario in which uninfected and infected ticks simultaneously feed on the same vertebrate host. In their original experiment, THOV-infected *Rhicipicephalus appendiculatus* adults (donors) and uninfected *R. appendiculatus* adults (recipients) were allowed to feed on guinea pigs either within the same capsule (Figure 1A) or in separate capsules (Figure 1B). To differentiate donors from recipients on each animal, donors were assigned a specified sex and recipients were assigned the opposite sex. After feeding to repletion, recipient ticks were screened for THOV by plaque assay. Interestingly, 76% of the adult recipients feeding in separate capsules from donors and 64% of those sharing the same capsule tested positive for THOV. The experiment was repeated using the same two-capsule approach, with uninfected *R. appendiculatus* larvae or nymphs serving as recipients. Ten days following engorgement for larvae, and twelve days for nymphs, recipient ticks were screened for THOV. All three pools of recipient larvae and 72% of individual nymphs were positive for virus. During both experiments, daily blood samples were collected from guinea pigs and screened for THOV by plaque assay. No viremia was detected at any time point. These results demonstrated that THOV transmission from infected to uninfected ticks can occur while ticks feed on a vertebrate host without detectable viremia. These findings introduced the concept of nonviremic co-feeding transmission of TBVs [3].

Initial evidence of nonviremic co-feeding transmission was based on work involving THOV, a virus of limited medical relevance, ultimately prompting reassessment of how vertebrate hosts without detectable viremia contribute to the transmission and maintenance of TBVs of public health importance. In 1993, Labuda et al. [8] assessed nonviremic co-feeding transmission of tick-borne encephalitis virus (TBEV), which is responsible for several thousand human cases annually [9]. These studies utilized a two capsule infestation method, similar to that used by Jones et al. [3,8]. Eight guinea pigs were each infested with six TBEV-infected *Ixodes ricinus* adult females (donors) confined to one capsule (Figure 1B). Of these, four guinea pigs were simultaneously infested with 100 uninfected *I. ricinus* nymphs (recipients; 50 per capsule), and four were infested with 100 uninfected *R. appendiculatus* nymphs (recipients; 50 per capsule) (Figure 1B). An additional five guinea pigs were co-infested with six TBEV-infected *R. appendiculatus* adult female donors and 100 uninfected *R. appendiculatus* recipient nymphs (50 per capsule). Blood samples collected from each guinea pig at 4 days post-tick infestation were used to determine viremia status. Upon completion of tick feeding, all ticks and blood samples were screened for TBEV by plaque titration. Three guinea pigs (two with *I. ricinus* donors and one with *R. appendiculatus* donors) were found to have no infected donor ticks in actuality and were subsequently removed from the analysis. Of the remaining 10 guinea pigs, six developed detectable viremias at the single time point screened. Among recipient ticks fed on nonviremic guinea pigs, the mean infection prevalence was 41.1%. No significant differences in TBEV acquisition were observed between the two recipient tick species co-fed with *I. ricinus* donors, regardless of the viremia status of the guinea pigs. Similarly, vector efficiency did not differ significantly between the two donor tick species when *R. appendiculatus* nymphs served as the recipients. Lastly, infection prevalence of recipient nymphs was compared between the two capsules. On viremic animals, significantly more recipients became infected when feeding in the same capsule as the donors, whereas on nonviremic animals no significant difference was detected between the two capsules [8].

While the early work of Jones et al. and Labuda et al. was foundational in demonstrating co-feeding transmission of TBVs [3,8], certain methodological considerations should be noted when interpreting these studies. For example, Labuda et al. [8] assessed host viremia at a single time point (4 days post infestation) and concluded that hosts remained nonviremic during tick co-feeding. Because ixodid ticks feed for several days, more frequent sampling throughout the multi-day feeding period would strengthen confirmation of nonviremic conditions in such experiments [8]. Additionally, these early studies focused on co-feeding transmission involving hard ticks, whose prolonged feeding period may influence transmission dynamics differently than soft ticks, which feed for shorter durations.

The first study to directly examine co-feeding transmission of TBVs using soft ticks was published in 2004, when Lawrie et al. [10] infested seven mice with West Nile virus (WNV)-infected *Ornithodoros moubata* third instar nymphs (donors) and uninfected *O. moubata* second instar nymphs (recipients) allowing them to co-feed to repletion (<24 h). Five days after engorgement, 66 of the second instar nymph recipients were screened for WNV by nested reverse transcriptase–polymerase chain reaction (RT-PCR), and 22.7% (15/66) tested positive for WNV RNA. The remaining 15 s instar nymph recipients were screened after they had molted to third instar nymphs (45 days post engorgement), with 26.7% (4/15) testing positive for WNV RNA. Fifteen days after infestation, all of the mice were euthanized, necropsied, and their brains were screened by immunofluorescence assay (IFA) and RT-PCR. None of the mouse brains tested positive for WNV RNA by RT-PCR or WNV antigen by IFA. The negative data from the vertebrate host brains, along with the ticks being in contact with the mice for less than 24 h, provide evidence of nonviremic co-feeding transmission of WNV [10].

Additional studies evaluating co-feeding transmission of TBVs are summarized in Table 1 [3,8,10,11,12,13,14,15,16,17,18,19,20,21,22,23,24,25,26,27,28,29,30,31,32,33,34,35,36,37]. Many of these studies effectively utilize xenodiagnostic approaches, whereby naïve ticks are allowed to feed on a host previously exposed to infected ticks to assess transmission in the absence of detectable viremia. These studies encompass TBVs from five different virus families and include those transmitted by both hard and soft ticks. A range of donor and recipient tick species and life cycle stage combinations have been examined, along with a variety of vertebrate host species, including wild animals. These studies span from 1987 to the present day and reflect the ongoing effort to understand various aspects of co-feeding transmission of TBVs (Table 1).

### 2.1. Co-Feeding Transmission of TBVs on Virus-Immune Hosts

Jones and Nuttall investigated the role of virus-immune hosts in co-feeding transmission of TBVs using virus-immunized guinea pigs [24]. Prior to tick infestation, guinea pigs were immunized with two to three subcutaneous inoculations of THOV over a 14-day period. To confirm antibody titers, blood samples were collected by cardiac puncture at 21 days post-inoculation and screened by plaque reduction neutralization assay. Guinea pigs were then infested with THOV-infected *R. appendiculatus* adults (donors) and uninfected *R. appendiculatus* nymphs (recipients), with each life cycle stage placed in a separate capsule. Donor and recipient ticks were co-fed until the recipient nymphs were engorged (6–7 days post-attachment). Among the 100 recipients that co-fed on THOV-immunized guinea pigs, only one tested positive for THOV by plaque assay. In contrast, 80% (64/80) of recipients that co-fed on non-immunized guinea pigs tested positive for THOV. While these findings suggest that virus-immune hosts may limit nonviremic co-feeding transmission of TBVs, other studies have reported contrasting results [24].

In 1997, Labuda et al. [35] assessed tick co-feeding transmission of TBEV using *Apodemus flavicollis* (yellow-necked field mice) and *Clethrionomys glareolus* (bank voles) immunized either by subcutaneous inoculation of TBEV or by feeding of TBEV-infected *I. ricinus*. Tick co-feeding experiments were performed 7 weeks after immunization. Two TBEV-infected *I. ricinus* adult females (donors) and 20 uninfected *I. ricinus* nymphs (recipients) were placed in one capsule, while an additional 20 uninfected *I. ricinus* nymphs (recipients) were placed in a second capsule. Ticks were allowed to feed for three days, at which time hosts were euthanized and the ticks were collected. Serum samples collected prior to terminating tick co-feeding from all immunized hosts were positive for TBEV-specific antibodies by plaque reduction neutralization assay. Blood collected at euthanasia (3 days post tick attachment) was tested for viremia by plaque assay, and negative blood samples were further tested by intracranial inoculation of 1–2-day-old mice. Hosts immunized by TBEV-infected tick feeding did not develop detectable viremia, whereas at least one individual of each host species immunized by subcutaneous inoculation of TBEV did. Plaque assays of recipient ticks revealed that a greater percentage had become infected when co-fed on subcutaneously immunized and nonimmune *A. flavicollis* compared to *C. glareolus*. However, on hosts immunized by infected tick feeding, a higher proportion of recipients tested positive for TBEV after co-feeding on immunized *C. glareolus* compared to *A. flavicollis*. Moreover, for both host species, immunization by tick feeding was more effective at suppressing virus transmission between co-feeding ticks, yielding substantially fewer infected recipients than immunization by subcutaneous inoculation [35]. Regardless of host species, the efficiency of co-feeding transmission depended on the feeding proximity of donors and recipients: the prevalence of TBEV was higher in recipient nymphs feeding in the same capsule as donors than in those feeding in a separate capsule [35]. Overall, these findings suggest that co-feeding transmission of TBVs can occur on virus-immune hosts, but that both the route of immunization and the spatial proximity of donors and recipients influence transmission efficiency.

### 2.2. Co-Feeding Transmission of TBVs on Tick-Resistant Hosts

Acquired tick resistance (ATR) is a phenomenon in which a vertebrate host develops immunity to ticks after repeated tick exposure. It is frequently observed in non-natural vertebrate hosts (e.g., laboratory animals such as rabbits and guinea pigs), but not in wild host species that play important roles in the natural life cycle of ticks. ATR was first described in 1939 by William Trager, who repeatedly exposed guinea pigs, rabbits, and deer mice to ticks and monitored the number and weight of engorged ticks from each infestation as a measure of the host immune response [38]. Trager’s work demonstrated that non-natural hosts can mount a strong immune response against tick salivary components following repeated tick infestations, ultimately impairing tick feeding during subsequent exposures [38].

This seminal work led others to investigate how tick-resistant hosts influence viral transmission during co-feeding. Jones and Nuttall used previously tick-exposed guinea pigs to examine the impact of ATR on co-feeding transmission of THOV [20]. THOV-infected *R. appendiculatus* adults (donors) were co-fed with uninfected *R. appendiculatus* nymphs (recipients) on tick-resistant guinea pigs until the nymphs reached repletion. A reduction in the engorgement weights of both donors and recipients was observed, as well as a decrease in the number of recipient nymphs with detectable levels of THOV (15/116, 13%). Conversely, when donor and recipient ticks fed on tick-naïve guinea pigs, both engorgement weights and prevalence of THOV in recipient ticks increased (149/180, 83%). These findings indicate that co-feeding transmission of TBVs is substantially reduced on hosts with ATR and suggest that inducing host immunity to tick infestations may represent a potential strategy for controlling co-feeding transmission of TBVs [20].

### 2.3. TBVs with No Evidence of Co-Feeding Transmission

Some studies have shown that nonviremic co-feeding transmission of TBVs does not occur for certain viruses. In 2020, Pereira De Oliveira et al. [36] demonstrated that nonviremic co-feeding transmission of African Swine Fever Virus (ASFV), the only known DNA TBV, by the soft tick, *O. moubata*, does not occur on naïve pigs. Two chambers, each containing ASFV-infected *O. moubata* adults (donors) and uninfected *O. moubata* first instar nymphs (recipients), were placed on the abdomen of the same naïve pig for 3 h. After feeding, ticks were sorted into engorged and non-engorged groups. As a positive control, a third group of uninfected *O. moubata* nymphs were allowed to feed on the same experimental pig once it became viremic, confirming that nymphs can acquire ASFV via an infectious blood meal. Acquisition of virus was assessed by real-time PCR detection of the ASFV VP72 gene in individual DNA extracts from engorged recipients. None of the recipients tested positive for ASFV DNA, indicating that nonviremic co-feeding transmission of ASFV did not occur between infected adults and uninfected recipients [36].

The 2020 work by Pereira De Oliveira et al. [36] was not the first to report such findings. In 1993, Labuda et al. [33] examined several vertebrate species that serve as natural tick hosts to evaluate their ability to support co-feeding transmission of TBEV. The study demonstrated nonviremic co-feeding transmission of TBEV on vertebrate host species, such as *A. flavicollis*, as well as co-feeding transmission of TBEV on host species that developed viremia (e.g., *C. glareolus* and *Pitymys subterraneus*, pine vole). Conversely, other vertebrate species, such as *Phasianus colchicus* (ring-necked pheasant) and *Erinaceus europaeus* (European hedgehog), were largely resistant to infection and did not support co-feeding transmission of TBEV, although ticks were still able to successfully feed on these hosts [33]. Additional studies that did not find evidence supporting co-feeding transmission of TBVs include those by Gilbert et al. and Kazimírová et al. [25,26]. When viewed in conjunction with the findings by Pereira De Oliveira et al. [36] and Labuda et al. [33], these studies examining co-feeding transmission of Louping ill virus between different life stages of *I. ricinus* on wild host species and Yellow Fever virus between *I. ricinus* on Balb/c mice, respectively, indicate that recipient tick acquisition of virus during co-feeding depends on the specific combination of virus, tick species, and vertebrate host [25,26,33,36]. These findings highlight the need for further research assessing co-feeding transmission across a broad range of viruses, vectors, and vertebrate host species. Such work may help define the conditions required for co-feeding transmission of TBVs and clarify how this process occurs even in the absence of host viremia.

## 3. Time and Space as Requirements for TBV Transmission Between Co-Feeding Ticks in Nature

For co-feeding to occur, ticks must exhibit synchrony in their seasonal questing patterns and co-occur on the same vertebrate host (i.e., shared host preference). Nonaka et al. [39] demonstrated the importance of synchrony in tick seasonal questing patterns using mathematical modeling to evaluate how tick seasonality influences the relative contributions of three transmission routes (systemic, vertical, and co-feeding) in sustaining Powassan virus (POWV) in nature. In their model, systemic transmission was defined as the transfer of the virus between a vertebrate host and a tick through feeding on a viremic host; vertical transmission as the transfer of the virus from adult female ticks to her eggs; and co-feeding transmission as the transfer of the virus between infected and uninfected ticks feeding in close proximity on nonviremic vertebrate hosts. The model incorporated seasonal variation in tick activity and was updated monthly to reflect changes in tick feeding behavior and transmission dynamics associated with fluctuating tick and vertebrate host densities. Model simulations were run until the tick population reached a stable annual density cycle, after which each transmission route was selectively disabled while the rates of the remaining routes were varied. When larval and nymphal activity overlapped, POWV was predicted to persist in the tick population at or above observed levels, even in the absence of vertical or systemic transmission. Conversely, when co-feeding transmission was disabled under these conditions, POWV was unable to persist, suggesting that co-feeding transmission is the dominant route when larval and nymphal stages overlap. When these tick life cycle stages did not overlap, POWV persistence required both co-feeding transmission (between the same life cycle stages) and vertical transmission [39]. These results highlight the critical role of seasonal synchrony in enabling overlap of tick life cycle stages and facilitating co-feeding transmission of TBVs.

More broadly, the relative importance of systemic versus co-feeding transmission is likely determined by a combination of virus-specific, host-specific, and ecological factors. These include determinants of vertebrate host competence, such as the magnitude and duration of viremia, as well as ecological variables such as host population dynamics and differences in host-seeking behavior among tick species and their resulting patterns of host infestation. For some TBVs, it has been proposed that systemic transmission may be limited when viremia is insufficient in magnitude and/or duration to sustain efficient virus acquisition by feeding ticks, thereby increasing the relative importance of nonviremic co-feeding transmission [1,40]. While these dynamics are not fully defined for many TBVs, including POWV, they highlight the need to consider how both host factors and ecological factors shape TBV transmission routes in nature.

Although modeling studies underscore the importance of seasonal synchrony of tick life cycle stages in supporting co-feeding transmission of TBVs, field studies are required to confirm whether such dynamics occur in nature. Randolph et al. [41] investigated patterns of tick infestation on rodents in TBEV-endemic regions of Slovakia to assess the incidence of co-feeding between larvae and nymphs. Tick and small mammal abundance was highest between April and October, corresponding to the seasonal limits of tick activity. Tick infestation intensity was high, with a single host infested with as many as 242 ticks. *Dermacentor reticulatus* and *I. ricinus* were the most abundant tick species feeding on small mammals, with *C. glareolus* and *A. flavicollis* comprising the majority of small mammals sampled. Seasonal infestation patterns of *I. ricinus* and *D. reticulatus* on these hosts were analyzed to determine when larvae and nymphs fed concurrently. Overlap of *I. ricinus* larvae and nymphs occurred between April and July, whereas *D. reticulatus* larvae and nymphs overlapped from July to August. These time frames were then used to examine tick infestation patterns at the individual host level. For both tick species, larval and nymphal burdens on these hosts were highly aggregated, with approximately 20% of hosts supporting ~75% of ticks, a distribution consistent with the “80/20 rule” [41,42].

While the work by Randolph et al. [41] highlighted how larval and nymphal infestations are distributed among hosts, Bournez et al. [43] extended these findings by evaluating seasonal and annual variation in epidemiological parameters that enable *I. ricinus* nymph-to-larva transmission of TBEV in Alsace, France, over a three-year period (2012–2014). Each year was divided into three questing seasons: early April to early May, early June to early July, and early September to early October. Questing tick densities were measured monthly. Overall, seasonal density pattern reflected the main periods of activity for each life cycle stage: nymphs and adults peaked from April/May to early July, while larvae were active from April to October with a peak from May to early July. This pattern remained consistent across all three years, such that synchronous activity between tick life cycle stages provided opportunities for nymph-to-larva co-feeding transmission of TBEV. Small mammal abundance and the prevalence of larval/nymphal co-infestation were also assessed over the same period. In 2012, small mammal abundance was highest, yet larval/nymphal co-infestation prevalence was only 2.7%. Conversely, in 2013, small mammal abundance was lowest, but larval/nymphal co-infestation prevalence increased to 57.8%. When considered alongside overall questing tick density (low in 2012, high in 2013), these findings suggest that nymph-to-larva co-feeding transmission of TBEV is favored under conditions of high questing tick density and low vertebrate host abundance [43]. Collectively, these studies demonstrate how tick seasonality, host availability, and infestation patterns create opportunities for co-feeding transmission of TBVs to occur in nature.

Despite these favorable ecological conditions, experimental studies often report relatively high rates of virus acquisition among co-feeding ticks. Thus, it is important to consider how these findings relate to natural systems. In field settings, the prevalence of TBVs in tick populations is typically low, frequently less than 1% for viruses such as TBEV [44,45]. In addition, infection prevalence in ticks may vary over time following feeding or molting, potentially reflecting changes in viral load or detectability over time [32]. As a result, experiments that assess ticks shortly after feeding or molting may report higher apparent infection rates than those observed in nature. Although these dynamics remain incompletely characterized, they highlight the need for caution when extrapolating experimental co-feeding transmission efficiencies to natural TBV transmission cycles.

## 4. Role of Localized Skin Infection in Tick Co-Feeding Transmission

### 4.1. Saliva-Assisted Transmission (SAT) of Tick-Borne Viruses

Tick feeding is accompanied by the secretion of saliva, which contains a complex mixture of bioactive molecules that facilitate blood feeding and modulate host immune responses at the tick feeding site. These salivary factors possess anti-hemostatic, anti-complement, anti-inflammatory, and immunomodulatory properties that enable ticks to evade host defenses and successfully feed over multiple days, or in some cases weeks. Saliva-assisted transmission (SAT) is defined as “the indirect promotion of arthropod-borne pathogen transmission via the actions of arthropod saliva molecules on the vertebrate host” [7]. SAT was first demonstrated experimentally in studies investigating transmission of THOV by *R. appendiculatus*. In these experiments, guinea pigs inoculated with THOV together with salivary gland extract (SGE) from partially fed ticks exhibited significantly enhanced virus acquisition by feeding ticks compared to guinea pigs inoculated with virus alone [46]. Additionally, viremia was not detected in any of the tested guinea pigs. This study ultimately provided the first evidence that factors present in tick saliva could promote viral transmission independent of systemic vertebrate host viremia. Subsequent work extended these observations to additional viruses, including TBEV and POWV, demonstrating that tick SGE can enhance viral transmission and dissemination [47,48].

The mechanisms underlying SAT are thought to involve localized immunomodulation at the tick feeding site. Tick saliva is known to be capable of suppressing host inflammatory responses, inhibiting complement activation, modulating cytokine and chemokine signaling, and altering the function of immune cells [5,6,49,50]. Collectively, the effects of tick saliva create a locally immunomodulated microenvironment in the skin that can facilitate virus replication during tick feeding and subsequent viral dissemination. These effects are also relevant to nonviremic co-feeding transmission, whereby tick salivary molecules shape the cutaneous microenvironment in ways that facilitate localized viral replication and subsequent acquisition by nearby co-feeding ticks. Together, these interactions highlight the importance of the tick feeding site as a critical interface where tick saliva, virus replication, and host immune responses converge to influence transmission dynamics.

### 4.2. Localized Viral Infection at the Tick Feeding Site

Following the establishment of a saliva-mediated immunomodulatory environment at the tick feeding site, viral transmission during tick co-feeding relies on the vertebrate host serving as a bridge between infected and uninfected ticks. However, the cutaneous immunological mechanisms operating at the tick co-feeding site remain poorly defined. In 1996, Labuda et al. [34] investigated the effect of a localized skin infection in co-feeding transmission of TBEV. Using wild *A. flavicollis* and *C. glareolus* together with *I. ricinus* to mimic natural transmission conditions, the authors performed multiple co-feeding experiments to evaluate host viremia status, infection at the tick bite site, and viral acquisition by recipient ticks [34]. Regardless of host viremia, recipient ticks became infected only when TBEV was detectable in the skin at their feeding site, suggesting that localized skin infection is a critical component of co-feeding transmission of TBVs [34].

More recent studies by Obellianne et al. [27] further examined nonviremic co-feeding transmission using POWV-infected *Ixodes scapularis* female adults (donors) and uninfected *Haemaphysalis longicornis* nymphs (recipients). These experiments demonstrated that viral RNA was detectable at the tick feeding site but absent from distal skin sites, directly supporting earlier findings that co-feeding transmission is associated with localized viral infection at the tick co-feeding site [34]. In one of these experiments, a staggered (recipient-first) tick infestation strategy was employed, and daily blood samples were collected from each mouse to monitor viremia throughout the tick co-feeding period. q-RT-PCR screening revealed that some mice had no detectable viremia during the co-feeding period, yet recipient ticks that co-fed on these mice still acquired viral RNA, indicating that nonviremic co-feeding transmission of POWV occurred. In the nonviremic mice, viral RNA was detected in skin samples collected from the site of tick co-feeding but not in those taken from distal skin sites [27]. Together, these findings extend the observations of Labuda et al. [34] by providing additional evidence that localized viral infection at the tick feeding site is a central feature of nonviremic co-feeding transmission of TBVs.

### 4.3. Cellular Targets and Immune Responses at the Feeding Site

**Figure 2:**
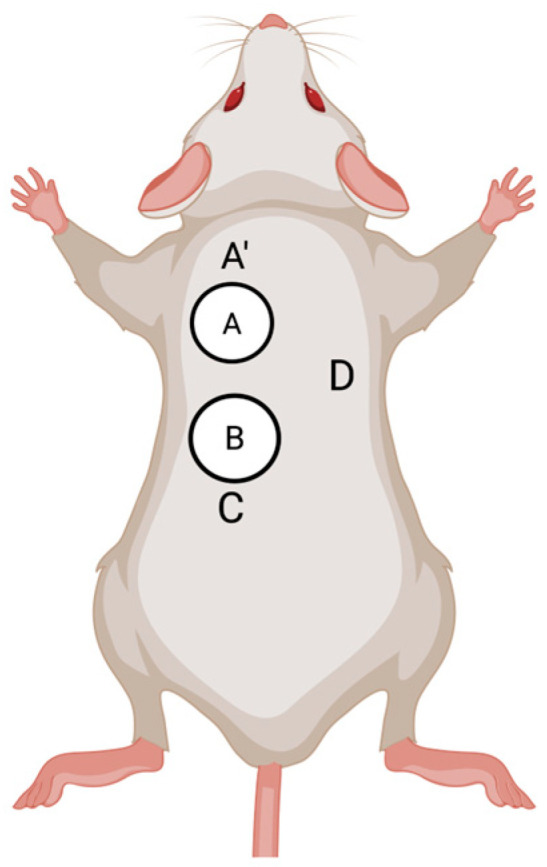
Location of skin explants harvested from mice in experiments conducted by [34]. White circles represent tick feeding capsules. Each letter represents the location from which a skin explant was taken. A and B represent tick infested skin sites. A’, C, and D represent uninfested (tick-free) skin sites at various distances from the tick infested skin sites. Created with Biorender.com (https://app.biorender.com/citation/699dd52e5828f89fd1ad314e) (accessed 22 March 2026).

To examine the cutaneous immunological mechanisms underlying co-feeding transmission, Labuda et al. [34] investigated cutaneous cells associated with virus replication and transmission by assessing both virus production and emigrating cells from skin explants collected from the site of tick co-feeding. After 24 h of incubation, TBEV was detected in skin explants from tick infestation sites (Figure 2, sites A and B) and from an adjacent uninfested site (Figure 2, site A’), but not from the uninfested site located furthest from the donor ticks (Figure 2, site C). These findings support the hypothesis that localized skin infection at the tick feeding site is important for co-feeding transmission of TBVs. Furthermore, two-color immunocytochemistry of cells emigrating from the skin explants revealed co-localization of TBEV antigen with MHC II-positive cells, recognized as Langerhans cells, as well as with neutrophils. However, because two-colored immunocytochemistry allows detection of only two markers simultaneously, it does not permit definitive identification of the specific cell types infected by TBEV in the skin.

Since the seminal observations by Labuda et al. [34], additional studies have further characterized the initial cell targets of TBVs at the tick feeding site. Immunofluorescence analysis at the feeding site of a single POWV-infected tick detected macrophages and fibroblasts that stained positive for POWV antigen at 3, 6, 12, and 24 h post-infection [51]. Similarly, immunohistochemical analysis at the feeding sites of TBEV-infected ticks revealed that after 3 h of tick attachment, TBEV antigen localized to fibroblasts and mononuclear cells, suggesting that these cell types could be initial targets of flavivirus infection [51,52]. In addition to immunohistochemistry, RNA in situ hybridization (RNA ISH) has also been used to provide insight into the localization of viral RNA at the tick feeding site. RNA ISH performed at 24 h post-attachment at the feeding site of individual POWV-infected ticks revealed co-localization of viral RNA with several *Mus musculus* target transcripts, including F4/80, CD11c, Vimentin, Keratin 14, and CD3ε. These findings indicate that various cell types (e.g., macrophages, dendritic cells, fibroblasts, keratinocytes, and T cells) may be early targets of POWV within the first hours of tick feeding [53]. Collectively, these studies provide important insight into the initial cell targets of tick-borne flaviviruses at the tick feeding site; however, because these investigations were conducted using single-tick feeding models rather than co-feeding systems, the fate of these infected cells and their role in facilitating viral transmission between co-feeding ticks remain unclear.

Based on the detection of TBEV antigen in migratory Langerhans cells derived from the tick co-feeding site, Labuda et al. [34] proposed a model in which infected Langerhans cells migrate to draining lymph nodes and subsequently present viral antigen to T lymphocytes [54,55]. Upon activation, T lymphocytes recirculate and can home to inflamed tissues, such as the skin site of tick co-feeding; however, it remains unclear what role these T lymphocytes may play in the context of viral transmission between co-feeding ticks and ultimately in viral dissemination from the tick feeding site. Thus, while this model provides a useful framework, the precise mechanisms by which TBVs are transferred between co-feeding ticks have yet to be fully elucidated.

## 5. Future Directions for Research on Co-Feeding Transmission of TBVs

Although co-feeding transmission of TBVs is well-established and recognized as a major contributor to viral maintenance in nature, the mechanisms facilitating this transmission route remain unclear. Advances in technologies, experimental tools, and in vivo models over the past decades provide new opportunities to delineate the mechanistic basis of co-feeding transmission. Namely, a major priority for the field is to integrate in vivo tick co-feeding models with high resolution single-cell and spatial transcriptomic approaches as well as advanced imaging techniques to characterize and track viral movement between co-feeding ticks. Single-cell RNA sequencing and spatial transcriptomics, coupled with flow cytometry and immunohistochemistry-based approaches, can be used to map the cellular composition, activation states, and spatial organization of immune cells within the cutaneous microenvironment during tick co-feeding. Additionally, multiphoton intravital imaging offers the potential to visualize viral dissemination and immune cell trafficking in real-time within the skin during tick feeding. Complementary immunological perturbation strategies in the vertebrate host, such as immune cell depletion, disruption of immune cell trafficking, and targeted inhibition of signaling pathways, will provide functional insights into the roles of specific immune cells and molecular pathways involved in co-feeding transmission of TBVs. Together, these approaches will enable a more comprehensive understanding of the cellular and molecular mechanisms governing co-feeding transmission of TBVs and will be critical for identifying novel targets for intervention.

## Figures and Tables

**Table 1:** Summary of all studies assessing co-feeding transmission of tick-borne viruses. NS = not stated.

Virus Common Name (Abbreviation)	Virus Species	Virus Strain/Isolate	Donor Tick Species—Life Stage	Recipient Tick Species—Life Stage	Host Species (Common Name)	Host Viremia Status During Tick Co-Feeding	Host Serological Conversion	Did Recipient Ticks Acquire Virus? (Y/N)	Reference
**SOFT TICKS (ARGASIDAE)**									
***Asfarviridae***									
African swine fever virus (ASFV)	*Asfivirus haemorrhagiae*	Liv13/33: isolated from *O. moubata*	*Ornithodoros moubata*—adults	*Ornithodoros moubata*—1st nymphal instar	*Sus scrofa domesticus* (Large White pig)	Viremia was not assessed.	Serological conversion was not assessed.	No	[36]
***Flaviviridae***									
West Nile Virus (WNV)	*Orthoflavivirus nilense*	NY99 isolated from *Phoenicopterus chilensis* (Chilean flamingo)	*Ornithodoros moubata*—3rd instar	*Ornithodoros moubata*—2nd instar (before & after molting)	*Mus musculus*—BALB/c	Viremia was not assessed.	Serological conversion was not assessed.	Yes	[10]
**HARD TICKS (IXODIDAE)**									
***Flaviviridae***									
Tick-borne encephalitis virus (TBEV)	*Orthoflavivirus encephalitidis*	Isolate 198: isolated from *I. ricinus*	*Ixodes ricinus*—adults	*Ixodes ricinus*—adults, nymphs	*Apodemus flavicollis* (yellow-necked field mouse), *Clethrionomys glareolus* (bank vole)	Viremia was detected by plaque assay of blood from hosts immunized by subcutaneous inoculation of TBEV and nonimmune control hosts. Viremia was not detected in hosts immunized by a bite of an infected tick.	Serological conversion was detected by plaque reduction neutralization test of serum samples taken prior to termination of tick co-feeding.	Yes	[35]
		Isolate 198: as noted above	*Ixodes ricinus*—adults	*Ixodes ricinus*—adults, nymphs	*Apodemus flavicollis* (yellow-necked field mouse), *Clethrionomys glareolus* (bank vole)	Viremia was detected by plaque assay.	Serological conversion was not assessed.	Yes	[34]
		Isolate 198: as noted above	*Ixodes ricinus*—adults	*Ixodes ricinus*—nymphs	*Apodemus agrarius* (striped field mouse), *Clethrionomys glareolus* (bank vole), *Pitymys subterraneus* (pine vole)	Viremia was not assessed.	Serological conversion was not assessed.	Yes	[33]
					*Apodemus flavicollis* (yellow-necked field mouse)			Yes	
					*Phasianus colchicus* (pheasant)			No	
					*Erinaceus europaeus* (hedgehog)			No	
		Isolate 9001 and 198: isolated from *I. ricinus*	*Ixodes ricinus*—adults	*Ixodes ricinus*—adults, nymphs	*Cavia porcellus* (guinea pig)	Viremia was not assessed.	Serological conversion was not assessed.	Yes	[8]
			*Ixodes ricinus*—adults	*Rhipicephalus appendiculatus*—nymphs				Yes	
		Isolate 198: as noted above	*Rhipicephalus appendiculatus*—adults	*Rhipicephalus appendiculatus*—adults, nymphs				Yes	
		pGGVsH, IC-D67G, IC-E112G, IC-D277A	*Ixodes ricinus*—adult females	*Ixodes ricinus*—nymphs	*Mus musculus*—BALB/c	Viremia was not assessed.	Serological conversion was not assessed.	Yes	[31]
		Czech TBEV—Hypr: isolated from blood of a TBEV patient	*Ixodes ricinus*—adult females	*Ixodes ricinus*—adult males, nymphs, larvae	*Mus musculus*—BALB/c	Viremia was not assessed.	Serological conversion was not assessed.	Yes	[32]
		chimaeric and WT—Hypr and Vs (isolated from blood of a TBEV patient) strains	*Ixodes ricinus*—adult females	*Ixodes ricinus*—nymphs	*Mus musculus*—BALB/c	Viremia was not assessed.	Serological conversion was not assessed.	Yes	[30]
		Czech TBEV—Hypr: as noted above	*Ixodes ricinus*—adult females	*Ixodes ricinus*—nymphs	*Mus musculus*—BALB/c	Viremia was not assessed.	Serological conversion was not assessed.	Yes	[25]
		Hypr strain: as noted above	*Ixodes ricinus*—adult females	*Ixodes ricinus*—nymphs	*Mus musculus*—BALB/c	Viremia was not assessed.	Serological conversion was not assessed.	Yes	[29]
		Hypr strain: as noted above	*Ixodes ricinus*—adult females	*Haemaphysalis inermis*—nymphs	*Mus musculus*—BALB/c	Viremia was detected by plaque assay.	Serological conversion was not assessed.	Yes	[28]
			*Dermacentor reticulatus*—adult females					Yes	
Powassan virus (POWV)	*Orthoflavivirus powassanense*	Spooner strain isolated from *I. scapularis*	*Ixodes scapularis*—adults	*Haemaphysalis longicornis*—larvae, nymphs	*Mus musculus*—BALB/c	Viremia was detected by q-RT-PCR.	Serological conversion was not assessed.	Yes	[27]
Louping ill virus (LIV)	*Orthoflavivirus loupingi*	Isolated from *I. ricinus*	*Ixodes ricinus*—adults	*Ixodes ricinus*—nymphs	*Apodemus sylvaticus* (wood mouse), *Clethrionomys glareolus* (bank vole), *Microtus agrestis* (field vole)	Viremia was not detected by plaque assay.	Serological conversion was detected at 14 days post-tick attachment by antibody neutralization assay.	No	[26]
		Isolated from *I. ricinus*	*Ixodes ricinus*—adults	*Ixodes ricinus*—nymphs	*Cervus elaphus* (red deer)	Viremia was detected by plaque assay.	Serological conversion was not detected by hemagglutination inhibition antibody tests prior to tick infestation; however, by day 8 post-tick attachment, serological conversion was detected.	No	[22]
					*Oryctolagus cuniculus* (New Zealand white rabbit)	Viremia was not detected by plaque assay.	Serological conversion was not assessed.		
					*Lepus timidus* (mountain hare)	Viremia was detected by plaque assay in co-feeding experiments on virus-naïve hosts. Viremia was not assessed during co-feeding experiment on virus-immune hosts.	Serological conversion was not detected prior to tick co-feeding by virus neutralizing assays; however, at 14 days post-tick attachment, serological conversion was detected.	Yes	
Yellow fever virus (YFV)	*Orthoflavivirus flavi*	Yellow Fever 17D-204: vaccine strain derived from wild-type Asibi strain	*Ixodes ricinus*—adult females	*Ixodes ricinus*—adult males, nymphs	*Mus musculus*—BALB/c	Viremia was not assessed.	Serological conversion was not assessed.	No	[25]
		CYD-2 (Chimeric Yellow Fever 17D/Dengue-2 virus)							
***Orthomyxoviridae***									
Thogoto virus (THOV)	*Thogotovirus thogotoense*	Sicilian SiAr 126: isolated from *Rhipicephalus bursa*	*Amblyomma variegatum*—adults, nymphs	*Rhipicephalus appendiculatus*—nymphs	*Cavia porcellus* (guinea pig—Dunkin Hartley)	Viremia was not detected by plaque assay.	Serological conversion was not assessed.	Yes	[21]
			*Amblyomma variegatum*—adults, nymphs	*Amblyomma variegatum*—nymphs					
			*Rhipicephalus appendiculatus*—adults, nymphs	*Amblyomma variegatum*—nymphs					
			*Rhipicephalus appendiculatus*—adults, nymphs	*Rhipicephalus appendiculatus*—nymphs					
		prototype IIA (isolated from a pool of *Rhipicephalus* and *Boophilus decoloratus*) or Sicilian SiAr 126 (isolated from *Rhipicephalus bursa*)	*Rhipicephalus appendiculatus*—adults	*Rhipicephalus appendiculatus*—adults, nymphs, larvae	*Cavia porcellus* (guinea pig—Dunkin Hartley)	Viremia was not detected by plaque assay.	Serological conversion was not assessed.	Yes	[3]
		prototype IIA or Sicilian SiAr 126: as noted above	*Rhipicephalus appendiculatus*—adults	*Rhipicephalus appendiculatus*—nymphs	*Cavia porcellus* (guinea pig—Dunkin Hartley)	Viremia was not assessed.	Serological conversion was detected at 21 days post-inoculation by plaque neutralization assay.	Yes	[24]
		Sicilian SiAr 126: as noted above	*Rhipicephalus appendiculatus*—adults	*Rhipicephalus appendiculatus*—nymphs	*Cavia porcellus* (guinea pig—Dunkin Hartley)	Viremia was not detected by plaque assay.	Serological conversion was not assessed.	Yes	[20]
		Sicilian SiAr	*Rhipicephalus appendiculatus*—adults	*Rhipicephalus appendiculatus*—nymphs	*Cavia porcellus* (guinea pig—Dunkin Hartley)	Viremia was not detected by plaque assay.	Serological conversion was not detected at 14 days post-tick attachment by virus neutralization assays.	Yes	[23]
		SiAr/126/72	*Rhipicephalus appendiculatus*—nymphs	*Rhipicephalus appendiculatus*—larvae	*Mus musculus*—BALB/c and Mx1 + A2G	Viremia was not detected in A2G mice but was detected in BALB/c mice by plaque assay.	Serological conversion was not assessed.	Yes	[19]
		Kamigamo: isolated from *H. longicornis*	*Haemaphysalis longicornis*—adults	*Haemaphysalis longicornis*—nymphs (molted to adults before being tested)	*Mus musculus*—BALB/c	Viremia was not assessed.	Serological conversion was not assessed.	Yes	[37]
***Nairoviridae***									
Crimean-Congo hemorrhagic fever virus (CCHFV)	*Orthonairovirus haemorrhagiae*	HD49199: isolate from a CCHFV patient	*Hyalomma truncatum*—adults	*Hyalomma truncatum*—adults	*Oryctolagus cuniculus* (rabbit)	Viremia was not assessed.	Serological conversion was detected at 15 days post-infestation by direct ELISA.	Yes	[18]
		IbAr 10200: isolated from *H. anatolicum excavatum*	*Hyalomma impeltatum*—adults	*Hyalomma impeltatum*—nymphs, larvae	*Cavia porcellus* (guinea pig—Dunkin Hartley)	Viremia was not detected by plaque assay.	Serological conversion was detected at 21 days post-tick infestation by indirect immunofluorescence and ELISA.	Yes	[17]
			*Hyalomma truncatum*—adults	*Hyalomma truncatum*—nymphs, larvae					
			*Hyalomma truncatum*	*Hyalomma truncatum*—adults	*Cavia porcellus* (guinea pig—Dunkin Hartley)	Viremia was not assessed.	Serological conversion was detected 30 days after tick co-feeding by an indirect fluorescent antibody technique.	Yes	[16]
***Phenuviridae***									
Bhanja virus	*Bandavirus bhanjanagarense*	RV760: isolated from *D. marginatus*	*Dermacentor marginatus*—adults	*Dermacentor marginatus*—adults	*Mus musculus*—NS	Viremia was not assessed.	Serological conversion was not assessed.	Yes	[15]
			*Ixodes ricinus*—adults	*Rhipicephalus appendiculatus*—nymphs					
Palma virus	NS	Isolated from *H. punctata*	*Dermacentor marginatus*—adults	*Dermacentor marginatus*—adults					
			*Dermacentor marginatus*—adults	*Rhipicephalus sanguineus*—adults					
			*Dermacentor reticulatus*—adults	*Dermacentor reticulatus*—adults					
			*Ixodes ricinus*—adults	*Rhipicephalus appendiculatus*—nymphs					
Severe fever with thrombocytopenia syndrome virus (SFTSV)	*Bandavirus dabieense*	Wuhan: isolated from a SFTSV patient	*Haemaphysalis longicornis*—adults	*Haemaphysalis longicornis*—nymphs	*Mus musculus*—Kunming (KM)	Viremia was not assessed.	Serological conversion was not assessed.	Yes	[11]
Heartland virus (HRTV)	*Bandavirus heartlandense*	M12-66: isolated from *A. americanum*	*Amblyomma americanum*—nymphs	*Amblyomma americanum*—larvae molted to nymphs	*Oryctolagus cuniculus* (New Zealand white rabbit)	Viremia was not detected by RT-PCR.	Serological conversion was detected by plaque reduction neutralization test of host sera taken post co-feeding.	Yes	[12]
		MO-4: isolated from a HRTV patient	*Amblyomma americanum*—nymphs	*Amblyomma americanum*—larvae	*Mus musculus*—BALB/c	Viremia was not assessed in first co-feeding experiment. Viremia was not detected by q-RT-PCR during second co-feeding experiment.	Serological conversion was only detected in second co-feeding experiment by serum immunoassay from terminal blood samples taken after co-feeding.	Yes	[13]
			*Amblyomma americanum*—nymphs	*Haemaphysalis longicornis*—larvae, nymphs					
***Sedoreoviridae***									
Great Island Virus (GIV)	*Orbivirus magninsulae*	NS	*Ixodes uriae*	*Ixodes uriae*	*Uria aalge* (Common guillemot)	Viremia was not assessed.	Serological conversion was not assessed.	Yes	[14]

## Data Availability

No new data were created or analyzed in this study. Data sharing is not applicable to this article.

## Abbreviations

The following abbreviations are used in this manuscript:

TBVs	Tick-borne viruses
SAT	saliva-assisted transmission
THOV	Thogoto virus
TBEV	Tick-borne encephalitis virus
WNV	West Nile virus
RT-PCR	Reverse transcriptase polymerase chain reaction
IFA	Immunofluorescence assay
ATR	Acquired tick resistance
ASFV	African swine fever virus
POWV	Powassan virus
SGE	Salivary gland extract
RNA ISH	RNA in situ hybridization
